# Disseminated Armillifer armillatus Infestation: A Rare Cause of Acute Abdomen

**DOI:** 10.7759/cureus.15038

**Published:** 2021-05-15

**Authors:** Joseph Asemota, Joseph Talbet, Owen Igbinosa, Osato Igbinovia

**Affiliations:** 1 Internal Medicine, Howard University Hospital, Washington, DC, USA; 2 Medicine, University College Hospital, Ibadan, NGA; 3 Plastic and Reconstructive Surgery, Howard University Hospital, Washington, DC, USA; 4 Cancer Imaging, University of Hull, Hull, GBR; 5 Radiology, University of Benin Teaching Hospital, Benin City, NGA

**Keywords:** pentasomiasis, acute abdomen, armillifer armillatus

## Abstract

We report a case of an 80-year-old symptomatic female with severe visceral *Armillifer armillatus* infestation who presented with complaints of progressively worsening colicky abdominal pain with associated constipation and mild abdominal distension. Imaging workup demonstrated unique radiological features of the parasite including multiple curvilinear opacities, measuring approximately 3 to 6 mm in length, scattered in the lung fields, abdomen, pelvis, and inguinal region. Histologic examination of inguinal biopsies revealed enlarged lymph nodes containing several parasitic pseudocysts. She was managed conservatively and received antihelmintics, with subsequent uneventful recovery. This case emphasizes the importance of meticulous differential diagnoses formulation. In the right clinical scenario, pentastomiasis should be considered in the differential diagnoses of patients with imaging evidence of multiple organ lesions, as a high index of suspicion is needed for the diagnosis of this entity and will help to avoid unnecessary invasive management.

## Introduction

*Armillifer armillatus* infestation is a zoonotic parasitosis caused by the ingestion of infective ova of *Armillifer armillatus*. The definitive hosts are snakes and reptiles [1]. Intermediate hosts, including carnivorous mammals and humans, acquire infection when they prey upon infected hosts or come in contact with infective ova that are released into the environment through the secretions and feces of definitive hosts [2]. Those at increased risk of infection include veterinarians, snake handlers, or individuals who consume snake flesh, especially pythons [3]. Although human infestation by the nymphs of *Armillifer* (syn. *porocephalus*) *armillatus* is relatively unknown in the highly developed regions of the world, it is not so uncommon in underdeveloped and developing regions [4, 5]. In fact, most cases have been reported in West and Central Africa, parts of Asia, Latin America, and the Middle East. Cases reported in developed countries are often seen among immigrants from highly infested countries and long-term travelers [6]. Presented below is a case of human infection with *Armillifer armillatus* with typical roentgenographic findings.

## Case presentation

An 80-year-old African female with a medical history significant for degenerative joint and spine disease, presented at the outpatient clinic with complaints of progressively worsening abdominal pain and vomiting of two weeks duration. Abdominal pain was rated at 4/10 at onset and described as dull but constant, with no known aggravating or relieving factors. Abdominal pain however progressed, and at the time of presentation was described as colicky and rated at 6/10 in intensity. She also reported constipation that began five days prior to presentation with mild abdominal distention. She however endorsed passage of flatus. There was a positive history of associated intermittent fever with chills and rigors. No history of alcohol consumption or cigarette smoking. She had previously received empiric treatment for malaria without resolution of symptoms.

On examination, she was acutely ill-looking, mildly pale, a cyanosed, anicteric and mildly dehydrated. Blood pressure was 154/82 mmHg and pulse rate was 92 bpm. Abdominal examination revealed mild distension, and generalized tenderness. There were no findings suggestive of hepatosplenomegaly. Bowel sounds were hyperactive. A presumptive diagnosis of partial bowel obstruction was made and she was subsequently referred to the emergency department for acute management. In the emergency department, she was placed NPO (*nil per os*) and nasogastric tube decompression was initiated. She was commenced on intravenous rehydration and blood work for laboratory investigation was obtained. Plain radiographs were also obtained.

**Figure 1:**
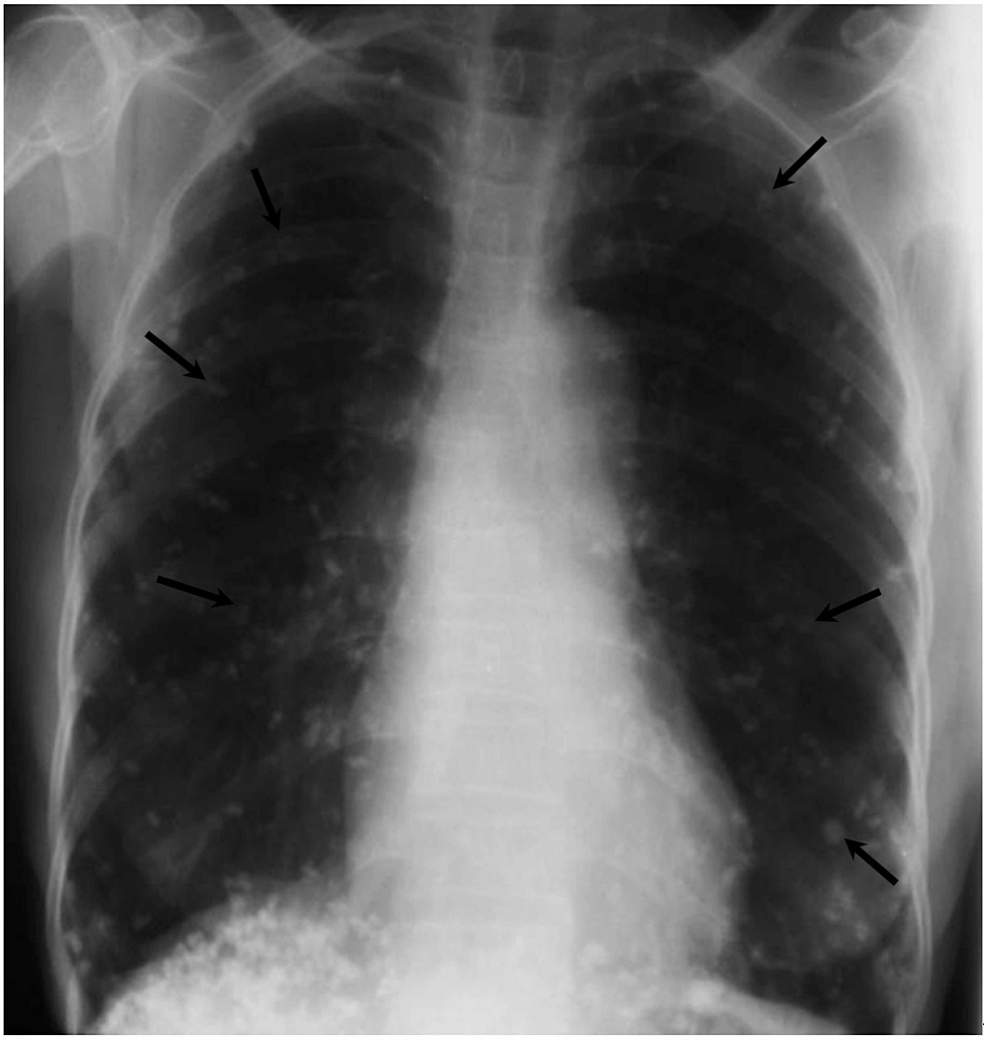
Postero-anterior chest radiograph showing numerous curvilinear calcific densities (arrows) scattered over both lung fields

**Figure 2:**
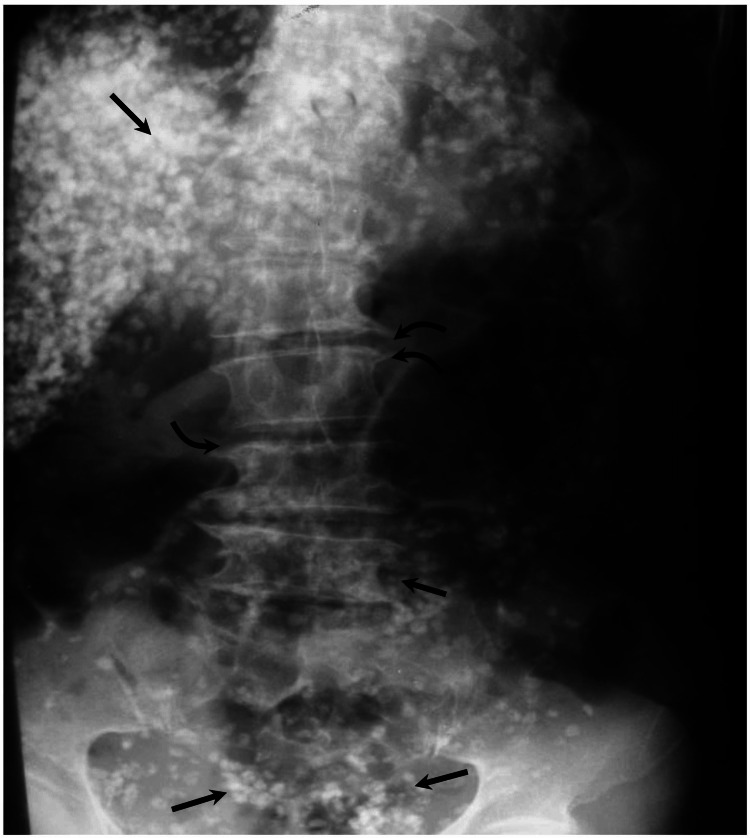
Plain abdominopelvic X-ray showing numerous crescentic calcifications (straight arrows) scattered throughout the abdomen and pelvis, spondylotic features are also present (curved arrows).

Results of laboratory investigations showed microcytic anemia (Hb: 9.8 g/dL; and mean corpuscular volume [MCV]: 73 fL) and eosinophilia. There was elevation in inflammatory markers (Erythrocyte sedimentation rate [ESR]: 31 mm/hr and C-reactive protein [CRP]: 16 mg/L). Liver function test revealed transaminitis (Alanine transaminase [ALT]: 92 IU/L and Aspartate transaminase [AST]: 87 IU/L) with only mild elevation in alkaline phosphatase (ALP) (146 IU/L) and gamma glutamyltransferase (GGT) (58 IU/L). International normalised ratio (INR) was within normal limits. A postero-anterior chest radiograph (Figure 1) demonstrated multiple curvilinear scattered opacities overlying both lung fields. These calcifications were also seen in the abdomen and pelvis films (Figure 2). They measured approximately 3 to 6 mm in length, although most were less than 4 mm. The shape of these calcific shadows varied from semicircular to rectilinear, with the majority presenting as intermediate forms.

**Figure 3:**
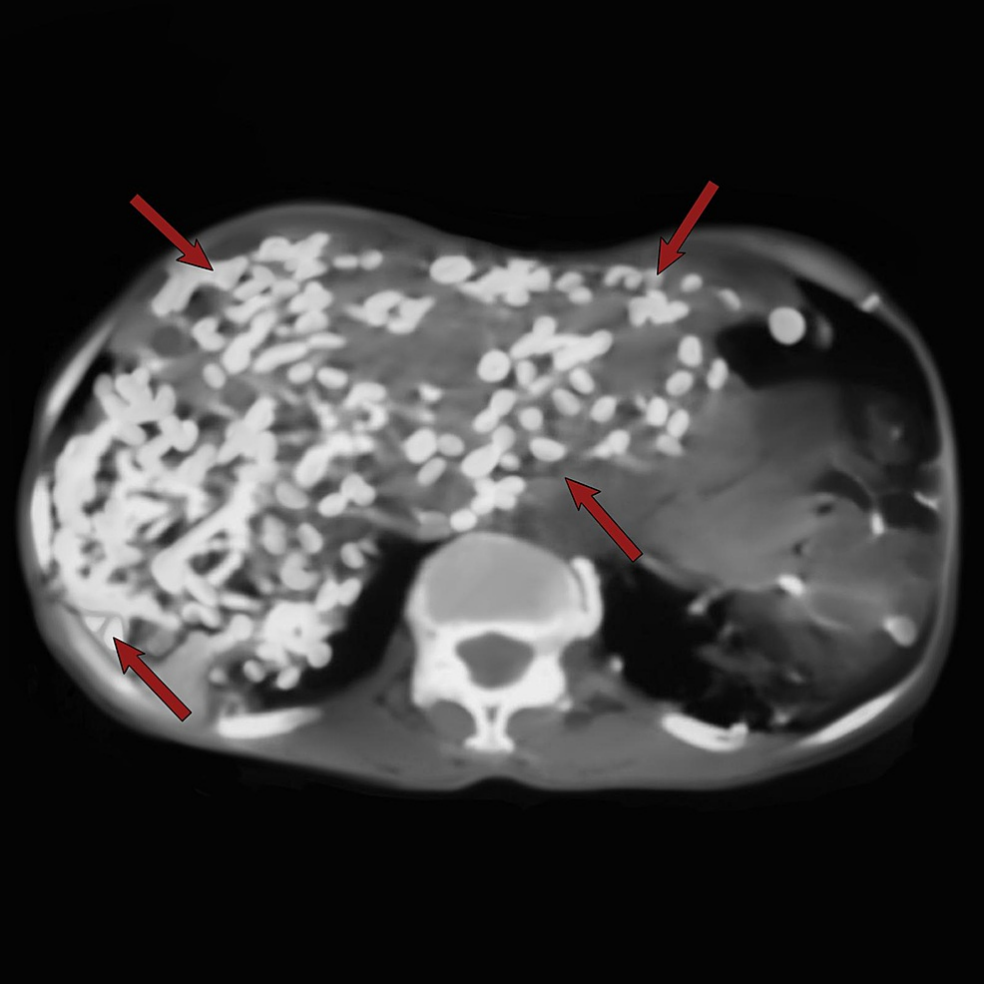
Axial non-contrast enhanced computerized tomography scan of the abdomen showing widespread infestation of the liver with Armillifer armillatus (red arrows).

**Figure 4:**
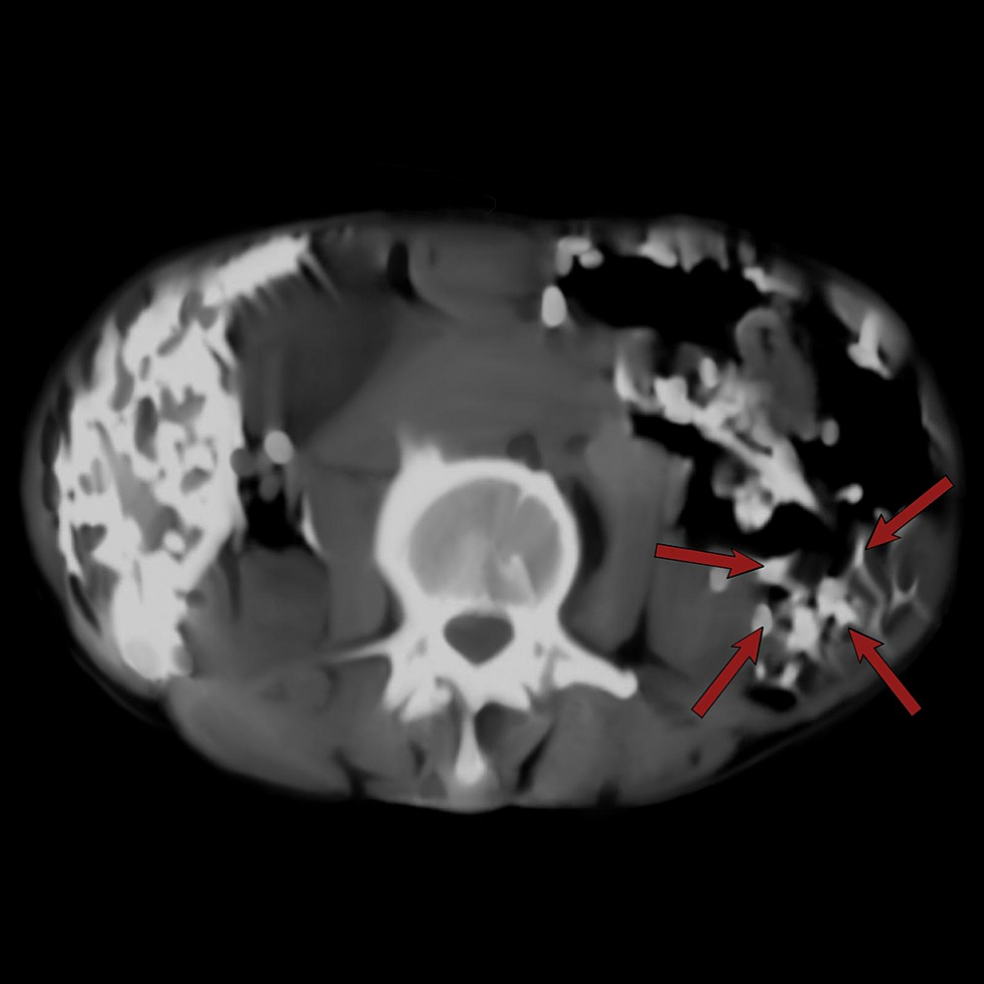
Axial computerized tomography scan of the abdomen showing infestation of the spleen with calcified nymphs of Armillifer armillatus (red arrows).

**Figure 5:**
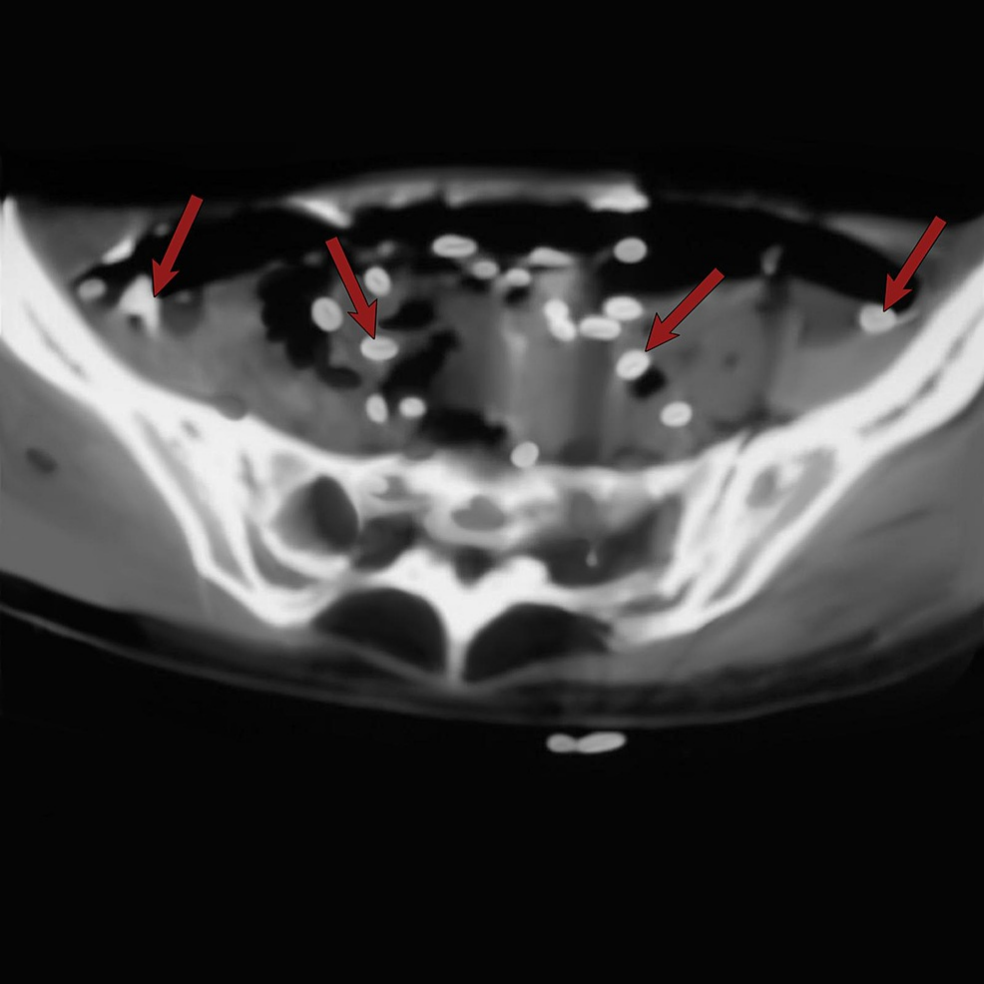
Axial non-contrast enhanced computerized tomography scan of the pelvis showing calcified Armillifer armillatus in the pelvis (red arrows).

The abdominal radiograph showed numerous crescentic calcifications scattered throughout the abdomen and pelvis, more numerous in the right and left upper quadrants and overlying the lumbar vertebrae. Some calcifications were noted in the inguinal regions bilaterally. Evidence of degenerative changes were present in the lumbosacral spine (Figure 2). Transabdominal ultrasonography was done and it showed numerous radio-opaque shadowing structures in the intraabdominal organs namely the liver, spleen, and both kidneys. Abdominal CT scan confirmed the heavy intra-abdominal organ infestation (Figures 3-5).

On interval history, the patient denied consumption of snakes or reptilian meat products. Image-guided transcutaneous biopsy of inguinal calcification was performed and histological examination revealed enlarged lymph nodes containing several parasitic pseudocysts. The parasites were diagnosed as encysted nymphs of *Armillifer armillatus*. She was managed conservatively and received treatment with antihelmintic agents. Acute symptoms subsequently resolved and the patient remained clinically stable.

In our patient, it was not quite possible to determine exactly how the infection was acquired as the patient denied ingestion of snakes or wild animals. It is however speculated that the patient may have unwittingly ingested drinking water or raw vegetables containing eggs of the parasite.

## Discussion

**Figure 6:**
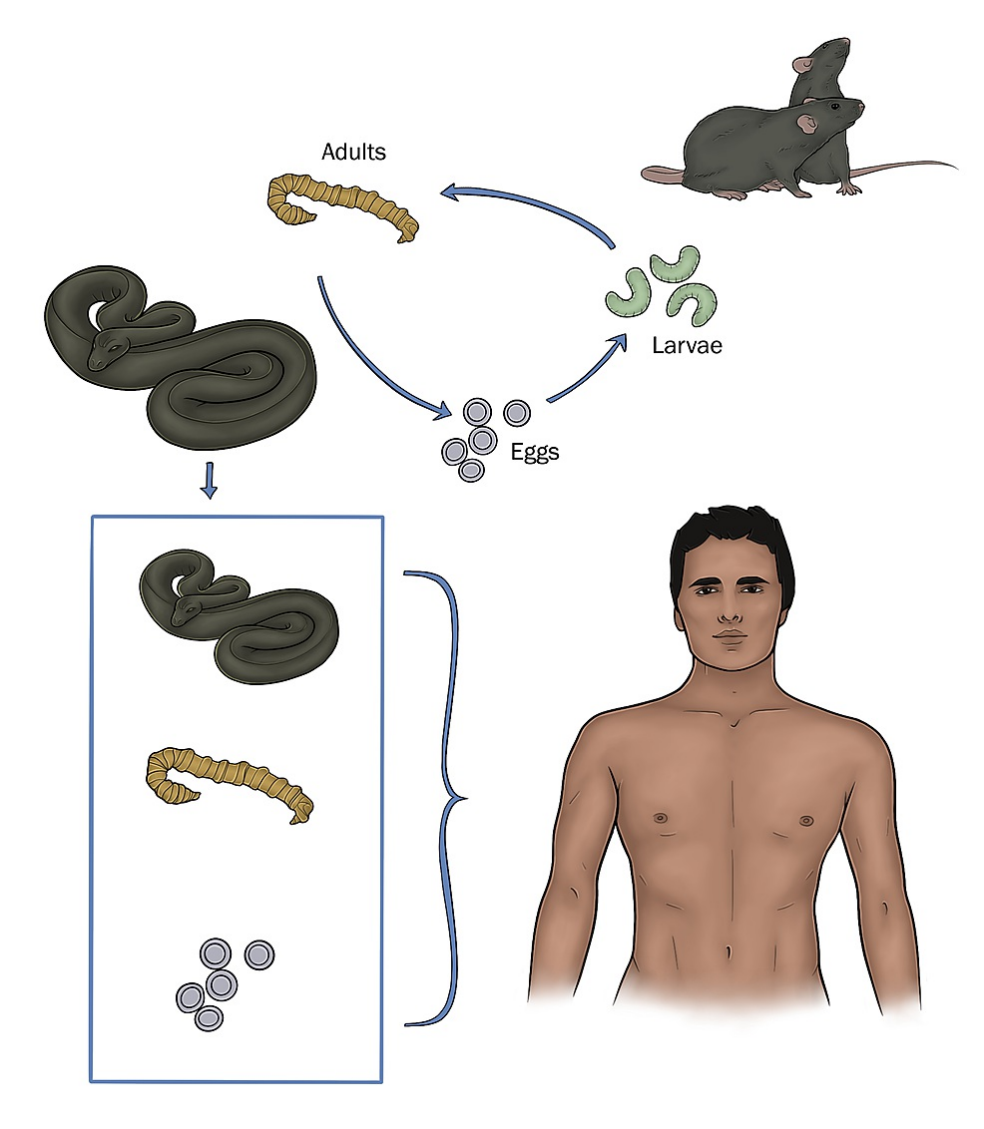
Summary life cycle of Armillifer armillatus.

*Armillifer* is a genus of obligate parasites that was first recognized as a human pathogen in 1847 [5]. The species of *Armillifer* which are parasitic in man and other animals belong to the family *Linguatulidae* ("tongue worms"), a group of worm-like, blood-sucking arthropods which are endoparasites of vertebrates [6]. They normally inhabit the respiratory tracts of snakes and reptiles and only occasionally infect humans [7]. The adult is whitish or yellow, and translucent with an elongated cylindrical vermiform body, tapering to a blunt tail [8]. The body is markedly annulated giving the worm a screw-like appearance, and has no trace of legs, antennae, or palpi. The mouth, surrounded by a chitinous ring, is situated at the anterior end of the "worm” with a pair of retractile hooks on either side of the mouth, which allow them to enter blood vessels and subsequently migrate to distant organ systems [7]. The sexes are distinct and in all species the male is much smaller than the female [8]. There are 13 genera and 43 species, of which five (*Porocephalus crotali*, *Porocephalus subulifer*, *Armillifer armillatus*, *Armillifer moniliformis*, and *Linguatula serrata*) are known to be possible parasites of man [9, 10]. Of these, *Armillifer armillatus* has been most frequently encountered and has been the subject of several articles describing the radiographic and other aspects of the disease [11-13]. The adult measuring about 250 µm in length, is found living in the trachea and lungs of pythons and other African snakes; the nymphal form is found in the lion, mandrill, giraffe, and African hedgehog, as well as in other wild and domestic animals in tropical Africa. The adult female lays double-shelled eggs resistant to water and gastric juice, in the posterior portions of the nasal passages of the snake. The eggs are subsequently passed with the bronchial secretions or with the excreta of the snake and embryonate upon reaching damp vegetation or water [4]. They are then ingested with contaminated water by various wild and domestic animals and occasionally by man [8]. Upon reaching the intestine of these intermediate hosts, the four-legged larvae are liberated from the egg and penetrate to the liver, lungs, mesenteric nodes, kidneys, and other organs. During development the larva molts several times and is finally encysted as a nymph [8]. When the animal which serves as the intermediate host is ingested, the nymphs are liberated and enter the lungs of the definitive host, where they develop into adult forms. In man, the cycle comes to an end and the larvae (nymphs) encyst in the liver, intestinal mucosa, lungs and peritoneal surfaces especially over the liver [8]. The nymph lies coiled within the cyst with its ventral surface corresponding to the convexity of the curve. This position is responsible for the characteristic radiographic appearance [11]. A summary of the life cycle of *Armillifer armillatus *and how individuals get infected is depicted in Figure 6 below.

In the majority of patients, there are few or no symptoms attributable to the infection and most patients are totally unaware that they harbor the parasites [5]. Most cases are discovered incidentally during autopsy or routine X-ray for other indications. Symptomatic patients may present with abdominal pain, persistent cough and night sweats [14]. However, in heavy infestation as seen in our patient, the migration of many live larvae beneath the peritoneum or pleura may cause sufficient irritation and pain to mimic an acute abdominal condition or pleurisy [7]. The majority of the larvae lie in the sub-peritoneal tissue, especially around the liver and spleen, or in the mesentery [11]. Pneumonitis, bronchitis, pleuritis, pericarditis, hepatitis and peritonitis have been noted in patients with severe infection and intestinal obstruction has occasionally been ascribed to fibrotic bands resulting from the dead cysts [11].

Diagnosis of visceral pentastomiasis is based on radiograph findings of calcified nymphs, or histopathological examination of biopsied lesions [3]. The calcified nymphs of Armillifer are usually easily recognized. They are almost always localized to the abdomen and chest and are rarely seen elsewhere. They are always multiple and are seen more often in the upper abdomen than in the lower abdomen or thorax. They are more commonly seen in the right upper quadrant, either on or below the peritoneal surface of the liver [13]. However, they may be seen as scattered calcifications anywhere in the peritoneal cavity around the spleen, mesentery, lungs and pleura, and even rarely in the scrotum. Their shape is distinctively crescentic, horseshoe or comma shaped, or coiled and vary from 4-8 mm in size [11]. Some may appear oval or rectilinear in outline. They are not found in muscle, which distinguishes these parasites from the calcified cysts of cysticercosis. If there is any doubt, a survey film of the muscles of the thighs or limbs will help to exclude cysticercosis. A typical calcification of the nymphs might be confused with calcified mesenteric lymph nodes, calculi, or even with malignancy especially in regions where infection is not prevalent. For example, Machado et al. reported an unusual case of visceral pentastomiasis that was resected as a primary hepatic neoplasm [15]. However, a useful hint is that the parasites are almost invariably multiple and scattered throughout the abdomen.

With respect to management, identified cases should be managed conservatively but symptomatic patients may benefit from antihelmintics such as mebendazole [1]. Surgical intervention may become necessary when parasitic infestation load becomes too large that it causes intestinal obstruction.

## Conclusions

In conclusion, it is important to entertain a broad range of differential diagnoses. In the right clinical context, a high index of suspicion should be maintained for possibilities such as pentastomiasis, especially in patients with imaging evidence of multiple organ lesions.
